# Reflexive Green Nationalism (RGN): A sociological antidote to the climate crisis?

**DOI:** 10.3389/fsoc.2022.1021641

**Published:** 2022-10-14

**Authors:** Lorenzo Posocco, Iarfhlaith Watson

**Affiliations:** School of Sociology, University College Dublin, Dublin, Ireland

**Keywords:** environmental sociology, climate change, nationalism, nation-state, global risks strategies, climate change strategies

## Abstract

What can theories of nationalism and the nation-state tell us about climate change? Much of the available literature, including works by prominent thinkers Ulrich Beck and Bruno Latour, identify it as a collective global challenge rather than a local and national one. But is it really so? This article develops an original theoretical framework integrating the theory of “reflexive modernity”, theories of nationalism, and case studies of green nation-states. The goal is to change the observation point and search for original solutions to the climate crisis. Building on this theoretical framework, this study puts forward the following claims: (1) climate change is undeniably a global phenomenon, but its causes are national. It can be traced back to a small number of top polluting nation-states (the US, China, Russia, India, Japan and EU28) whose historical share of carbon dioxide and other greenhouse gases, the main cause for global warming, surpasses 74%; (2) Most of these nation-states are entrenched in Resource Nationalism (RN), a form of nationalism that sees the environment as a resource to exploit; (3) there exist forms of sustainable nationalism, which this study conceptualizes as Reflexive Green Nationalism (RGN); (4) the solution to climate change is local rather than global. It depends on top polluters' capacity to re-modernize and develop RGN; and (5) according to the Intergovernmental Panel on Climate Change, if emissions are not reduced by 43% by 2030, the world is likely to cross the tipping point into a global climate catastrophe. Therefore, updating these nation-states and their ideology to more sustainable forms is humanity's best shot at halting the climate crisis.

## Introduction

The Intergovernmental Panel on Climate Change (IPCC) is the United Nations body for assessing the science related to climate change. In 2022, it consisted of “270 scientists from more than 60 different countries” who contributed to the most recent IPCC report (IPCC, 2022). The report is the latest of a number of denunciations of political immobility vis-à-vis the climate crisis and a serious warning that if no significant effort is made in the 2020–2030 decade to reduce carbon dioxide emissions and other greenhouse gases (GHG), the temperature of the planet will rise above the 1.5°C threshold of pre-industrial levels by 2040 (IPCC, 2022). This will set in motion a series of events related to changes in weather patterns resulting in extreme weather events, droughts, fires, rising sea levels, floods, and as a result, dramatic issues on the social level such as massive immigration and potentially a large number of dead.

It is clear that we are approaching an irreversible tipping point that jeopardizes human and non-human life on this planet (Conversi, 2020), and yet, the current historical conjuncture doesn't seem to provide the world with a favorable stage where the climate crisis can be successfully addressed. In fact, it poses two main problems. The first is that most countries are still recovering from the COVID-19 Pandemic and seem to focus more on shorter term goals such as recovering from the disruption brought by the virus, regaining economic competitiveness on the international stage, and restoring social life as it was before 2020. Moreover, other global risks are seen as more imminent than climate change, such as the threat of nuclear war stemming from the ongoing Russia-Ukraine conflict and/or the many other problems that this conflict brought, i.e., the increasing price of gas and oil, and problems in the export of wheat that are affecting most countries in the world, especially the poor.

It is true, climate change is a different global risk (Beck, 2008). Unlike pandemics, financial crises and wars, it is almost invisible and spreads over a longer period, two elements that make it a particularly insidious threat. And yet, IPCC scientists were unequivocal that a change of course is needed now, not after the Pandemic or the Russia-Ukraine conflict. To state it bluntly: if high-carbon emissions continue in the world, especially in top polluters such as China, the USA, Russia, EU28,^1^ and India, temperatures will rise above the 4°C threshold by 2100. To put things in perspective, the planet has not seen temperatures rising more than 2.5° in 3 million years (IPCC, 2022). Scientists have warned that we have a very short period, around 10 years, to drastically decrease the use of fossil fuels and implement a series of strategies that will reduce the human impact on the environment. This is not the place for details, which can be found in the 3,675 pages of the freely-downloadable IPCC report, but to summarize: if nothing changes, we will face the climate change worst case scenarios. An anthropogenic catastrophe of global proportion.

The second problem is that we live in a world system of nation-states dominated by nationalism (Brubaker, 2015; Malesevic, 2019). Nationalism is intended here as an ideology “entailing the belief that the world is naturally divided into nations that have distinctive cultural and physical characteristics inscribing them on the human landscape over time” (Posocco, 2022, p. 10). It is a highly anthropocentric ideology that puts humans before the environment, which is preponderantly inscribed in and perceived as a “part” of the nation-state. Hence expressions belonging to the realm of banal nationalism (Billig, 1995; Fox, 2008; Fox and Miller-Idriss, 2008; Skey, 2009) or everyday nationalism such as “national environment” or “national territory,” “national lakes,” “national mountains,” “national parks,” etc. There are many forms of nationalism, i.e. ethno-nationalism, populist nationalism, economic nationalism, and most of them see the environment as something to exploit, a “resource,”^2^ allegedly for the good of the nation, although “only a tiny minority of the population actually benefits from their extraction and exploitation” (Conversi, 2020, p. 630). However, there are also more critical and greener forms of nationalism that drive a small number of nation-states, such as Norway, Denmark, Sweden, Switzerland, and Germany, to protect their national environment and develop more sustainably (Conversi and Posocco, 2022).

While most of these forms of nationalism very often overlap and merge, they are all ideologies – the term ideology is to be understood here as a set of ideas that provides people with guidelines that are necessary to interact “with” and “in” the world (Althusser, 2001)–that lock up nation-states in themselves, “making them principally worry about matters of internal security, domestic homogeneity and national growth and less about global issues and other nations' troubles” (Posocco and Watson, 2022, p. 2). All national governments, green or not, acting on the international stage are predominantly driven by nationalism; their primary goal is to preserve the state and the nation, to maximize their security and their relative power position in relation to other nation-states (Waltz, 1979; Mearsheimer, 2001). This strongly impacts nation-states' capacity to cooperate and coordinate, let alone to stand in solidarity and support each other, which some have pointed to as key factors when it comes to global risks (Conversi, 2020; Eriksen, 2021).

For the sake of clarity, we know the science of climate change and how to address it. Scientists gave us the knowledge and the answers to most of the problems stemming from it, and we could start to fix them right now (Harvard Center for Climate, Health, and the Global Environment, 2021). What we don't know is how to make sure that the above-mentioned top-polluting nation-states, which also happen to be the most powerful nation-states on the planet, apply the required strategies in the window of time we have left. So far they haven't. Indeed, most of these nation-states lag, in terms of climate change performance (CCPI, 2022), dangerously behind other greener nation-states. This is where social science can help in understanding why some nation-states improve their management of the climate crisis while others do not. The analysis of the various forms of nationalism that drive nation-states helps us understand why some stick to Resource Nationalism (RN), an ideology at the core of which is the exploitation of the environment, while others develop more sustainable forms, such as Green Nationalism (GN) (Conversi, 2020; Conversi and Friis Hau, 2021; Conversi and Posocco, 2022; Posocco and Watson, 2022).

Attempting to contribute to an emerging body of literature that bridges nationalism studies and climate change, the main claim of this article is that, while climate change is undeniably a global phenomenon, its causes and solutions are national and can be found in a small number of top polluters. Making this claim is to confront numerous studies, including the work of Beck (2009) and Latour (2018), which argue that climate change is a collective global challenge and that other forms of polity and ideology, such as the cosmopolitan state and cosmopolitanism (Beck), and the terrestrial society and terrestrialism (Latour), are a better fit to address it.

**Figure 1:**
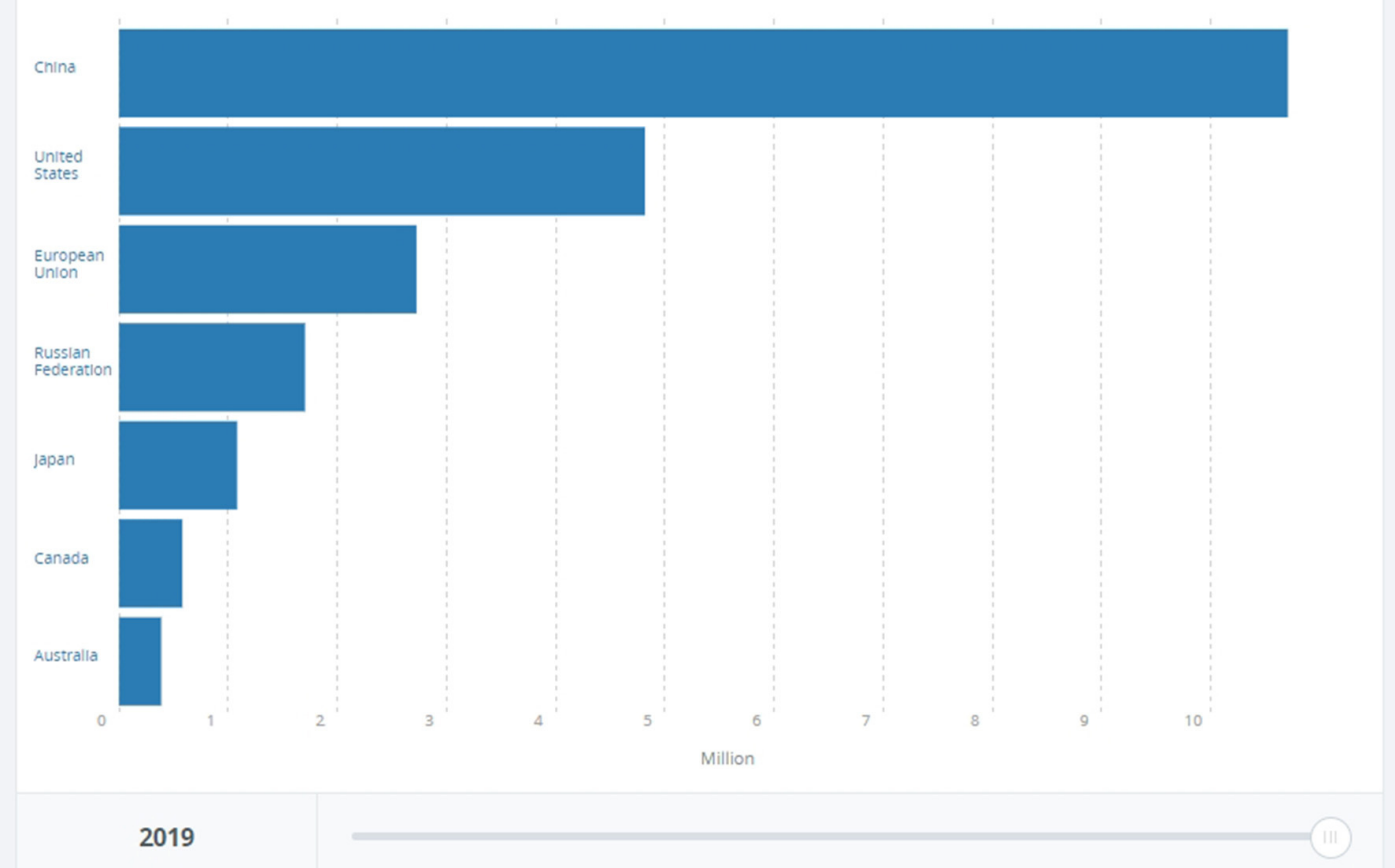
Share of CO2 emissions of China, the United States, EU28, Russian Federation, Japan, Canada, and Australia. Year: 2019. Data and graphic from the World Bank, at https://data.worldbank.org/indicator/EN.ATM.CO2E.KT?contextual=default&end=2019&locations=CN-US-EU-RU-JP-AU-CA&start=2019&view=bar (accessed August 9, 2022).

**Figure 2:**
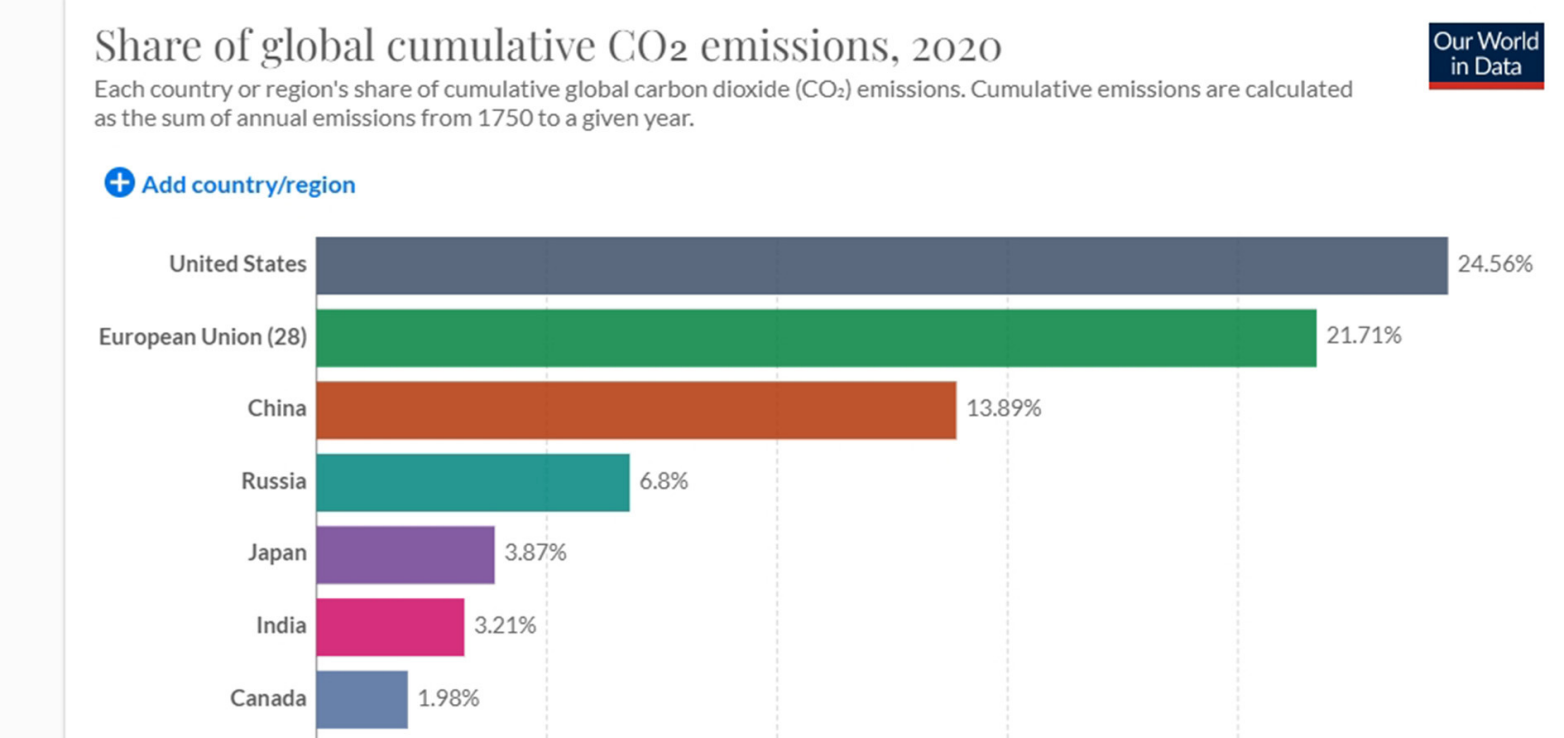
Historical share of carbon emissions of the US, EU28, China, Russia, Japan, India, Canada, Australia. Source Our World in Data, 2020, freely accessible at https://ourworldindata.org/.

**Figure 3:**
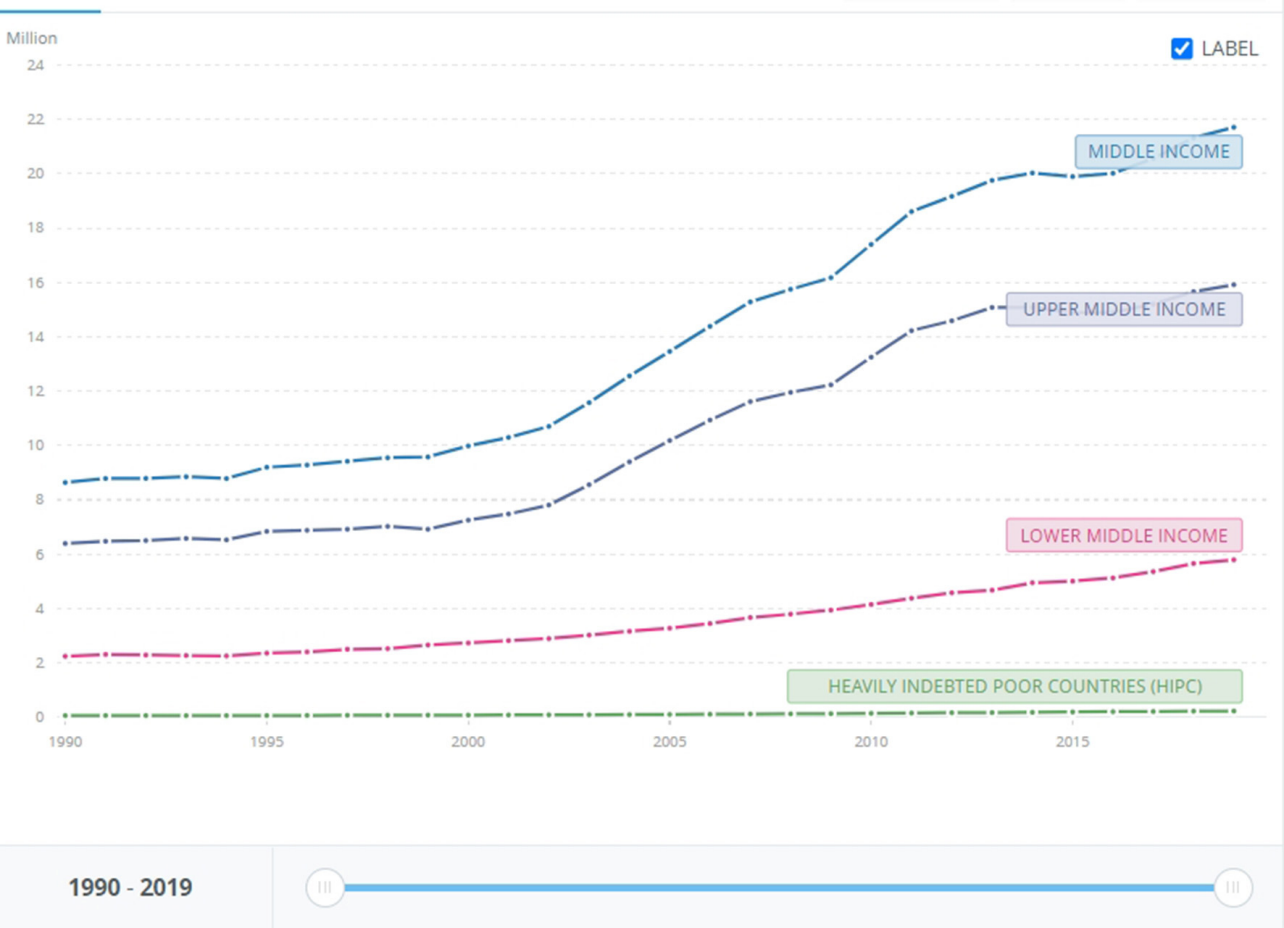
CO2 emissions in relation to countries' income worldwide. 1990-2019, Source: World Bank. At https://data.worldbank.org/indicator/EN.ATM.CO2E.KT?contextual=default&end=2019&locations=XP-XE-XT-XN&most_recent_year_desc=false&start=1990&view=chart (accessed August 9, 2022).

And yet, a focus on the national rather than global arenas is not without logic. In fact, it is a choice in line with risk-mitigation theory, which divides strategies into *unanimous, majoritarian* and *local* (Yudkowsky, 2008). While unanimous strategies are unworkable because they require the cooperation of all nation-states, and majoritarian strategies require decades to make most but not all nation-states agree on the solutions, local/national strategies require a much narrower, and more feasible, focus on a single or a few nation-states (Yudkowsky, 2008, p. 334). Especially after scholars acknowledged, en masse, the failure of global governance in triggering a global green transition (Goldin, 2013), focusing on single top polluters, i.e., the US, China, India, EU28, Japan, and Russia, (see Figures 1–3 below bringing evidence of these countries' CO2 emissions [Figure 1 shows cumulative emissions from 1750 to present; Figure 2 shows emissions from 1980 to present, and Figure 3 shows the level of emissions in 2019]) makes even more sense. Such focus shows that the main obstacle to resolve the climate crisis is the incapacity to shift from Resource Nationalism to Green Nationalism. So far, these nation-states have proven incapable of developing forms of nationalism that are more reflexive (Beck et al., 1994; Beck, 2016), thus self-critical and ready to implement strategies that would make them greener and less disruptive to the national and global environment. Hence the second claim of this article: if, as it seems, climate change is a problem that falls on the nation-state and overall on nationalism as the ideology that drives it, then there is an urgent need to trigger re-modernization and “green” nationalism. The questions are: what are the factors that prevent nation-states from re-modernizing and adopting green nationalism? Can we, and how do we, trigger it?

## Nation-state, nationalism and re-modernization vis-à-vis climate change

Before 2020, literature bridging nationalism studies and climate change was lacking, and there remains a dearth of such literature (Conversi and Posocco, 2022). This is surprising given that as early as 1986 Ulrich Beck pointed to the nation-state as one of the structural elements of modernity that contributed to giving birth to what he epitomized as “Risk Society” (Beck, 1986). Beck wasn't the only one recognizing the importance of the nation-state system vis-à-vis global commons. In the early 1990's, David Held was one of the first scholars pointing to the inadequacy of the nation-state system to manage global risks (Held, 1995). And yet, nobody posed the question of re-modernizing this system and what characteristics the new nation-state should possess. The same is true about nationalism, the ideology at the basis of the nation-state. Most studies focused on critique, looking at the elements of nationalism that make nation-states incompatible with the struggles against the climate crisis. Even Beck's posthumous book The Metamorphosis of the World Bank (2022) theorized the replacement of both the nation-state and nationalism with cosmopolitanism and cosmopolitan communities. The same is true for Latour, who in *Down to Earth* (2018) rejects the present system and ideologies, dismissing both local and global and, with them, the national and all it comprises (including political groups that he calls “the right and left”) to propose the concepts of terrestrial and terrestrialism, a society in which any human action must be subject to scrutiny vis-à-vis the impact that it has on the planet. While the latter is (obviously) a necessity, Latour doesn't say which institutions should supplant the existing ones, how to convince powerful oil business and corporations, entangled in a multi-layered web of interests of all sorts (economic, political, social, national and international), to stop drilling, mining companies to stop their disruptive extractive activities, farmers to stop intensive farming, chemical plant companies to stop dumping their waste into rivers, oceans and land, nuclear plant companies to stop producing nuclear waste, or top polluters, such as China, to convert immediately, and more fully, to the environmental creed.

Prasenjit Duara–perhaps the first, in 2020, to exhaustively outline the interrelations between global risks and nationalism at the annual conference organized by the Association for the Studies of Ethnicity and Nationalism (ASEN)–saw nationalism as the heart of all the crises in the modern world, but even he did not have concrete solutions beside appealing to a rather abstract concept of hope: “I conclude the essay not with a ready answer but with thoughts about why we cannot not function without the hope of human agency” (Duara, 2021, p. 620). Around the same time, in 2020, Daniele Conversi stressed the importance of connecting the field of nationalism studies to the phenomenon of climate change and identified a form of nationalism, green nationalism (GN), which could function as an alternative to resource nationalism (RN). The latter is characterized by the exploitation of nature by nation-states and by the denial that such exploitation is harmful to the planet. Forms of GN identified among minority nations such as Catalonia and Scotland, would involve an environmentally-focused agenda used by these stateless nations for their political, rather than environmental, goals (i.e., political autonomy). Another work by Conversi stressed the importance of what he called “exemplary ethical communities”: “human communities with a track record of sustainability related to forms of traditional knowledge and the capacity to survive outside the capitalist market and nation-state system” (Conversi, 2021, p. 5582). None of these studies saw the nation-state and nationalism as potentially suited to host forms of nationalism that are environmentally friendly at the larger national level.

Posocco and Watson (2022) were the first to tentatively outline such a potential in the nation-state and suggest that some nation-states already walked a greener path. They focused mainly on Germany, but pointed to the need for more investigations insofar as other nation-states were headed in the same direction. Their study provided evidence that, starting with the 1970's, mass mobilization on the part of civil society found an attentive listener, rather than an opponent, in the German state. This resulted in environmental policy sustained in the long run and the development of a tradition of environmentalism that today is half a century old (although the first glimpses of ecologism are to be found as far back as the second half of the 19th century). Nationalism and environmentalism intertwined to such an extent that, as Uekötter (2014) put it, the latter became a structural part of the national project. Another key development is that having a healthy national environment became something in which Germans found pride as a nation. Fuelled by environmental awards such as the EU's award for the greenest cities in Europe, and environmental rankings such as the Climate Change Performance Index (CCPI), this sentiment reinforced green nationalism in Germany, strengthening the German people's belief that caring for the environment is something good and worth pursuing.

Further studies by Conversi and Posocco (2022) focused on nation-states that scored very high in the IPCC and CCPI rankings: Germany, Sweden, Switzerland, Norway, and Denmark. They found that these countries share elements that made them more suitable than others to become greener. Particularly, they highlighted the following: (1) developments of ecologism and environmentalism rooted for a century or more, (2) the lock-in of environmentalism as an ideology shared at all levels of society, (3) free and effective environmental movements, (4) inclusivity and welfare, and (5) the bonding between nationalism and environmentalism leading to national pride in environmental achievements.

Such developments in the above-mentioned nation-states are directly tied to the upheaval brought by modernity in all spheres of society. Modernity is intended here as a new society developed first between the 18th−19th century in Western Europe, based on industrialization, urbanization, science and technology, slowly supplanting society based on religion, and the birth of the modern nation-state (Beck, 2008). While bringing positives, this society also gave birth to unintentional side-effects (Beck et al., 1994) such as air pollution, large amounts of waste, soil depletion, water pollution, and an increase in diseases due to globalization, urbanization, and population growth.

For the fathers of reflexive modernity, Beck et al. (1994), side-effects are essential to make societies aware of the problems they create, apply solutions and avoid catastrophes. For example, since the 19th century, ecologic movements grew in number and size as a reaction to the side-effects of modernity. Acknowledging the shortcomings of modern society, Marx developed “Das Kapital,” Ibsen conceptualized *Friluftsliv*, “a philosophical lifestyle based on experiences of the freedom in nature and the spiritual connectedness with the landscape” (Gelter, 2000, p. 78), Henry David Thoreau played a similar role in the US, while John Muir advocated for the creation of national parks that would protect large regions of the country from mining, drilling, and other forms of exploitation of nature. These are just a few examples of reactions by individuals who witnessed the side-effects of modernity on 19th century and early 20th century societies and worked, even before modernization theory, to re-modernize modern society.

Environmental movements born in the second half of the 20th century, which John McNeill identified as the starting point of the Great Acceleration toward anthropogenic climate change (McNeill, 2016), must be seen through the same lens, stemming from solid evidence and increasing general awareness that harm was being done to nature whose effects would last generations, and that a radical change of course was needed. Especially during what is generally recognized as the environmental turn in the 1960s-1970s, environmental policy increasingly regulated human-nature relations while the idea that nature is something to protect, not to exploit, locked in. Initially in the West, and later almost everywhere, voices rose to demand modernity to re-modernize. The reader might remember the first Earth Day on April 22, 1970, which gathered millions of people around the world and that today can leverage more than 1 billion people in more than 193 countries (Rome, 2010). And yet, the opportunities of a more sustainable society arising from “the bads” (Beck, 1999, p. 152) were only embraced by a restricted number of nation-states. Most others ignored the science and kept business as usual. Most of them are still stuck in outdated methods of modernization although valid alternatives exist, i.e., in terms of energy production and consumption, China is still highly dependent on coal.

This evidence casts grave doubts on modernization theory, thus on the reflexive capacity of modern nation-states to solve the problems that modernization projects create. The way the world is, or rather is not, dealing with climate change (but the same could be said about a number of other global risks, i.e., nuclear conflict, nuclear waste and loss of biological and cultural diversity) provides a host of evidence for this. Even those exemplary green nation-states (Conversi and Posocco, 2022) that historically are ranked very high in terms of climate-change performance have been warned, in the last CCPI report, that they too need to do more. The reason why some nation-states learned from their mistakes and attempted to re-modernize while others lagged (and still lag) behind depends mainly on the problems and obstacles of re-modernization.

## The problems of re-modernization

**Figure 4:**
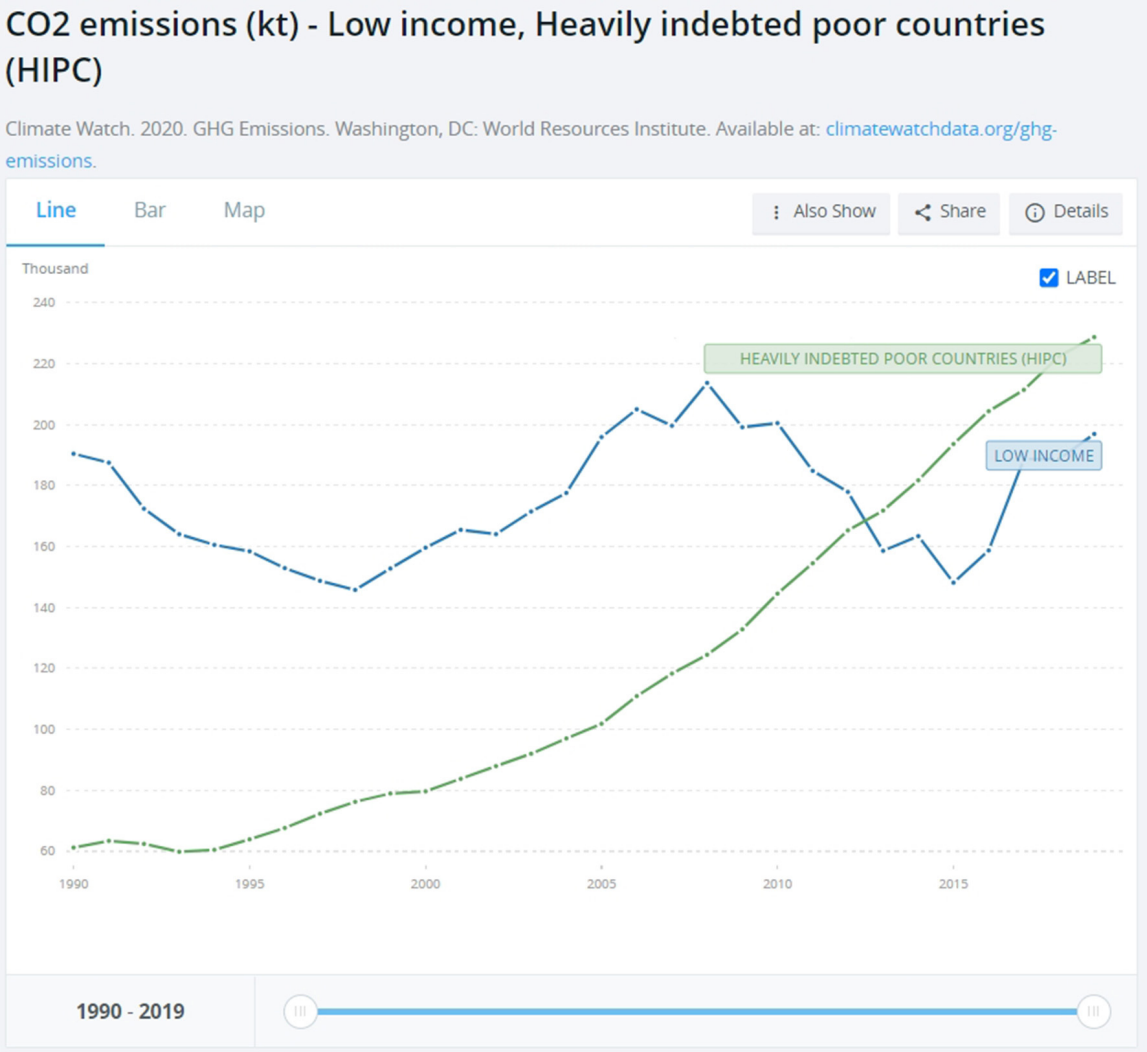
Share of CO2 emissions, low income and heavily independent poor countries. Notice the shift from Million to Thousand tons of carbon dioxide Year: 1990–2019. Source: World Bank at https://data.worldbank.org/indicator/EN.ATM.CO2E.KT?contextual=default&end=2019&locations=XM-XE&most_recent_year_desc=false&start=1990&view=chart (accessed August 10, 2022).

**Figure 5:**
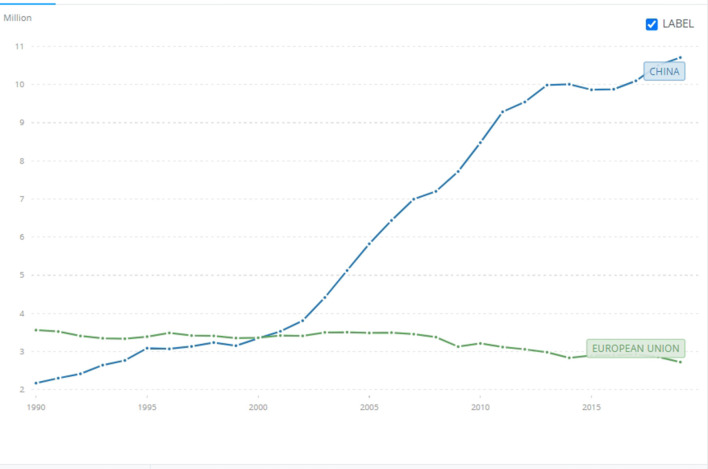
CO2 emissions. China and EU28 in comparison. Timeframe: 1990-2019. Source World Bank: https://data.worldbank.org/indicator/EN.ATM.CO2E.KT?contextual=default&end=2019&locations=CN-EU&most_recent_year_desc=false&start=1990&view=chart (accessed August 9, 2022).

Even a superficial look at modern industrialized nation-states such as the US, China, Russia, Japan, India, and EU28 shows that while climate change is a global problem, its causes are national. These 33 nation-states' historical share of carbon dioxide and other GHG emissions surpasses 74% (see Figure 2 below). Considering that the world is divided into 193 nation-states, the remaining 26% of carbon emissions is shared among 158 countries. A very straightforward equation shows that most of them, especially those with low income and the highly indebted poor countries (HIPC) (World Bank, 2022. It is worth noting that their emissions are measured in terms of thousand, not million, tons of carbon dioxide.), weighed in the range of 0.1–0.3% in comparison to top polluters (see Figure 4 below).^3^ Among these countries, poor nation-states such as Mozambique, Zimbabwe, Puerto Rico and Myanmar have no role at all in the climate crisis and yet, according to the Global Climate Risk Index (2021), climate change affects them the most (see Figure 3 below). Data from the World Bank shows that the above-mentioned top polluters are still emitting most of the carbon dioxide that endangers humanity (see Figure 1). Although it is important to make distinctions, i.e. China's emission are still rocketing while the one EU28 are, albeit slowly, decreasing (see Figure 5 below), not only is this small number of nation-states historically responsible for leading the world toward the climate catastrophe, but they are also not doing much, certainly not enough, to prevent it.

While China and India, both developing countries, have often justified their rocketing emissions as the unfortunate but necessary modernization efforts to compete with modern superpowers, the same justification doesn't apply to others who are fully modern and developed nation-states. This suggests there is no linear correlation between knowledge and re-modernization. Some nation-states do re-modernize, as is evident by the case studies of exemplary green nation-states such as Norway, Denmark, Sweden, Germany, and Switzerland, while others don't (Conversi and Posocco, 2022; Posocco and Watson, 2022). The latter linger in old patterns of modernization, entrenched in unsustainability. The reason is that many things can go wrong, and they do, on the road to re-modernization that block knowledge from triggering reflexivity and change. We know almost nothing about it, as we lack a systematic study of the problems of re-modernization. This section will attempt to scratch the surface of a potentially significant field of study that will hopefully be the focus of future research.

Cullenward and Victor (2021) identified in the “inconvenient problems of politics” (2021, p. 7) the core factor that determines the success or failure of climate policy. They see politics as a key element for making significant progress in reducing emissions. Similarly, we identify it as a key element for re-modernization. In fact, political systems define the processes through which nation-states' governments make official decisions for the nation, i.e., incentivize green technology or not, create what Cullenward and Victor (2021) call “effective market-based regulations” or opt for other types of climate regulations, outlaw mining and drilling or allow them, etc.

What's important here is that while top polluters, especially China, the US, Russia, and India, have political systems that are structured differently and are driven by different ideologies, some factors that they share are at the core of their failure in terms of re-modernization. Above all, politics in these nation-states end up serving the interests of a small number of powerful groups, which a solid body of literature has proven being linked to the exploitation of natural resources such as oil and gas, rather than the Aristotelian *to koinei sympheron* (the common interest). For example, according to The Global Wealth Report (2021), 110 Russian citizens control 35% of the total household wealth across the vast country, most of which is connected with natural resources such as gas and oil. Given this evidence it is not surprising that Russia is in 56th position out of 64 nation-states in terms of Climate Change Performance (CCPI, 2022), and in 126th position in the 2021 Democracy Index (DI, 2021). China is in 148th position, identified as an “authoritarian regime,” and India is in 46th position, a “flawed democracy.” The US shares the same label as India and is in 26th position. While the US is ranked much higher than China and relatively higher than India, a study by Gilens and Page (2014) casts stark shadows over it. They analyzed 1,779 recent policy outcomes and found that economic elites and organized groups representing the interests of businesses linked to environmental exploitation (i.e., oil companies, gas companies, chemical companies) have a major influence on government policy (around 78%), while average citizens have little or no independent influence (around 5%).

Corporate interest, personal profit, and corruption entering the political arena of top polluters seem to be a key factor hindering re-modernization. In fact, these countries were unable to adjust their political systems to better address these and related environmental problems. They lacked reflexive governance, as Feindt and Weiland (2018, p. 662) put it, or institutional reflexivity, as Giddens (1991) defined it. They lacked ‘the regularized use of knowledge about circumstances of social life as a constitutive element in its organization and transformation' (Giddens, 1991, p. 20). Reflexive governance would have led them to a deeper ideological shift at the structural level, involving democratization, increased representativeness, inclusivity supported (among others) by effective environmental movements, and welfare, which a recent qualitative study on green nation-states identified as important factors facilitating the implementation of successful environmental policy (Conversi and Posocco, 2022). In Germany, Sweden, Switzerland, Norway and Denmark, mass mobilization and political reflexivity by social-democratic states led to environmental policy sustained in the long run.

Other studies suggest that a synergy between top-down and bottom-up forces (which is difficult to achieve in authoritarian systems such as China or poorly representative systems such as the US) seems to be a condition sine qua non for environmental success. Without it, even when governments attempted to regulate dirty industries, market and coordinative instruments (Jordan et al., 2003), to implement environmental policy (Lenschow, 2002) and integrate stakeholder participation (Feindt and Newig, 2005), they had very little success (Feindt and Weiland, 2018). Their strategies did not have a significant impact on the activities that generate climate change. This happens because, even when knowledge is institutionally reflexive–informed by science and political power (Giddens, 1994)–numerous other factors (discussed below) can, and do, hinder or slow down re-modernization.

## Not only politics. Other obstacles to re-modernization

Powerful deniers might (and do, according to Klein, 2020) fight even harder when they think that governments will implement climate policy and negatively affect their interests. Another example that takes into account micro-sociological dynamics comes from an Italian case study. In 2019, Italy's government acknowledged the necessity of incentives toward green mobility and construction, and made 60 million euro available toward these sectors. An investigation led by Italian newspaper *Libero* (Free), later confirmed by the Italian judiciary, on 22 big car dealers in Italy found that, as soon as the season of incentives started, they all increased prices. To summarize, an electric or hybrid car was more affordable before incentives than after. There was also the “diesel dupe” scandal regarding the German automobile industry, particularly involving Volkswagen cars sold to foreign markets. The EPA (US's Environmental Protection Agency) found that Volkswagen had intentionally programmed diesel cars to only activate their emissions controls during laboratory emissions while when driving they emitted 40 times more nitrogen oxides that contribute to the formation of smog and acid rain, as well as affecting tropospheric ozone (BBC, 2015). The marketing strategy of Volkswagen was to sell the idea that diesel cars were as green as the others. This would allow them to keep selling them, thus making money, without investing in a new expensive productive chain manufacturing less polluting vehicles. While in both the Italian and German case there have been governmental investigations, what this evidence tells us is that knowledge and governmental action are not sufficient to re-modernize, and that the road toward re-modernization is filled with obstacles even when governments decide to take it. In this perspective, Bruno Latour was right when he argued that awareness (or knowledge) is one thing, but control is quite another matter (Latour, 2018).

Another issue is that, besides producing enormous capabilities to re-modernize, knowledge is also a potential source of great destabilization (Giddens and Pierson, 1998). Everywhere, knowledge of climate change has been a source of deep fractures and clashes within and between supporters and deniers. Among others, these clashes have materialized in the killing of numerous environmental activists, the latest of which, at time of writing, are Dom Phillips and Bruno Pereira, murdered in the extreme west of the Amazonas.^4^ Many studies point to indigenous communities in Colombia, Ecuador, Peru, Bolivia, Venezuela, and Brazil, as well as countries in the Asian hemisphere and on the African continent being approached by corporations and states interested in their territories. Building a new dam, increasing the national food production or extracting raw material are some recurring causes of wars that indigenous communities around the world fight, usually against much stronger opponents. These communities are systematically under attack, and according to a study by Amnesty International (2022), their people are often treated as second-class citizens although they have a much more sophisticated knowledge of the natural world; their land, forests and biodiversity flourish; and they live sustainably creating barriers against climate change. So much so that Conversi (2021) epitomized these communities as “exemplary ethical communities,” living examples for a potential sustainable future in the Anthropocene.

Clearly, clashes do not occur just in remote areas of the world but also in the very centers of modern societies, within government coalitions and communities. One recent example involved the US Democratic Party. In December 2021, coal investor and Senator John Manchin stated that he opposed the Democratic Party's energy policy (his own party) (The Guardian, 2022), which was a blow to Joe Biden's Green New Deal, the democrats' answer to Trump's renowned denialism. The event is clear evidence of major economic and political interests behind the climate crisis and the social and political conflicts that the latter gives birth to.

Finally, another key problem of re-modernization is that re-modernization theory doesn't deal with the role of discursive constructs and misinformation in hindering re-modernization. The first has to do with how discourses on climate change can include or exclude people from participation in top-down or bottom-up processes (Lassen et al., 2011). The second has to do with a phenomenon that Levy called “bad thinking epidemic” (Levy, 2021): society's inability to create a healthy epistemic environment where people can distinguish between reliable and unreliable sources of information. The two are related insofar as successful discursive construction is crucial to (1) create more explicit and specific knowledge about climate change and (2) deliver political communication ‘to actors about actions on both a global and a local level' (Lassen et al., 2011, p. 425). When coming to mass mobilization – the environmental activism led by various environmental actors - the lack of such communication might (and does) result in vagueness creating tension in terms of “rationales, relevant participants, invited vs. self-organizing forms, when to involve, and context sensitivity” (Lassen et al., 2011, p. 425). Not knowing exactly what one could or should do is not the only problem. Misinformation affects people's knowledge of the climate crisis, as a result a consistent number of people deny it [phenomenon of climate change denial, see Brulle (2020)], underestimate its consequences and/or avoid taking the necessary countermeasures (i.e., enacting effective climate policy, greening infrastructure and production processes, adopting sustainable behaviors such as recycling, investing in green energy and transportation). For Levy (2021), the main point is the crisis of media, especially social media. He argues that the environment in which people are given and/or seek information is so wide, complex and fractured, where everything and the opposite of everything seems to be true, that those who do not have specific knowledge on, say, climate change, lose any point of reference. This process, which also involves the phenomenon of fake news (among many publications on the issue, The Misinformation Age by O'Connor and Weatheral (2019) and Latour's Down to Earth (2018) deeply and critically engage with it) is another piece (perhaps a relevant one) adding to the problem of re-modernization, as it gives way to numerous tensions, at all levels of society, slowing down or hindering it.

## Nationalist ideology, reflexivity, and re-modernization

Ideologies consist of beliefs, values, and judgements about the world that guide people through life, their interactions in society, with other societies and people and also with the natural world (Posocco and Watson, 2022). Changing governments' and people's ideas about and behaviors toward nature is, first and foremost, a matter of ideology. Latour's concept of a “Terrestrial” mode of living (Latour, 2018), which involves a total shift from viewing the planet as a universe “in” which humanity lives to a universe “with” which humanity develops, requires a change in ideology. The same is true for Ulrich Beck's concept of cosmopolitanism and cosmopolitan society (Beck, 2002, 2009). For changing the way we treat, or threaten, nature, the ideas, thus the ideology that drive our beliefs on and relationship with nature, must change.

And yet, we must face reality. We don't live in a cosmopolitan nor a terrestrial world, we live in a world where the nation-state is the dominant political reality (Brubaker, 2015) and nationalism is the dominant political ideology (Malesevic, 2019). Nationalism has deep and well-established roots that shaped, and keep shaping, people's subjectivities and their ideas about and behaviors toward their state and fellow countrymen as well as other states and peoples. Everybody is a national in a way that goes beyond individual consciousness. As Zizek (1989) put it, one of the key characteristics of ideology is that it runs ubiquitously through society and everybody is influenced even when they think they aren't. It is in this ubiquitous and pervasive sense that nationalism also shapes the complex array of relationships between the nation, the national territory and the national environment (Posocco and Watson, 2022). Indeed, most of the causes of climate change reside in the way national ideology drives nationals' ideas of and behaviors toward the environment. If this is true, we must update the most common form of nationalism, Resource Nationalism (RN), to Green Nationalism (GN) as quickly as possible.

Major sources of inspiration come from case studies of green nation-states (Sweden, Norway, Denmark, Germany, and Switzerland), countries that took the “green way,” which projected them to the top of both CCPI and IPCC rankings (Conversi and Posocco, 2022; Posocco and Watson, 2022). In these countries, the transition toward green nationalism was triggered by (1) evidence of the environmental risks that their modernization efforts created (i.e. too much CO2 emissions, poor air quality, bad waste management, problematic intensive farming, misuse of fertilizers in agriculture leading to soil depletion, etc.), (2) reflexivity (self criticism), (3) analyses of potential strategies and available technologies to resolve the problems, and (4) successful application of strategies. Although it is important to stress substantial differences between these “green” nation-states, i.e., Germany is characterized by a strong green party, whereas Scandinavian countries don't. For example, Norway doesn't have a strong green party. Its Climate Change Performance (see CCP Index)^5^ is due to a number of factors, the most important of which is perhaps the fact that Norway is an “actively inclusive” state (Dryzek et al., 2002). Inclusivity means that environmentalists' demand are not only accepted (thus included in government debates) and turned into policy, but the state is keen on anticipating them. In addition, social democratic influence is strong in countries such as Norway and Denmark, which provide environmental (and other) organizations with the necessary funding to carry out their work, including drafting policy for the government and making sure that the latter correctly implements it. Switzerland is yet a different type of state, dominated by cantons characterized by strong autonomy, each one of them with their constitution, legislature (parliament), government and courts. The fact that these countries differ in the way they develop a green society is neither negative nor positive. Indeed, this element suggests that the roads to greening are many and potentially very different.

And yet, in spite of manifest differences, evidence gathered from existing comparative analyses shows that these societies' capacity to be reflexive, thus “self critical” (Beck, 2008, p. 79), assess the risks and act to avoid real catastrophes, was all but secondary (Conversi and Posocco, 2022). Their ability to re-modernize, thus to become better versions of themselves, was directly proportional to their ability to think critically. In addition, the knowledge created from the work of reflexivity was first and foremost directed toward the nation-state, to ameliorate national standards, not the world's.^6^ This is why we decided to use the expression “reflexive green nationalism” and apply it to the form of national ideology that these nation-states developed, and that future nation-states might be inspired by.

## What is reflexive green nationalism and how do we trigger it?

With Reflexive Green Nationalism (RGN) we mean an ideology shared by large segments of a nation-state that make civil and political society increasingly self-critical of modernization efforts that disrupt the environment, endangering human and non-human life. RGN drifts away from Resource Nationalism (RN), the most common form of nationalism we discussed earlier in this paper, mostly a self-idolizing and uncritical form of nationalism entrenched in old models of modernization that disregard short and long term consequences on the national environment. Unlike RN, RGN is a form of nationalism that accepts and encourages critique and reflection on the risks that national modernization projects create, and facilitates the search for solutions.

The above-mentioned studies on nation-states that developed RGN showed that the candidates for the subject of the critique of society, key elements triggering reflexivity and thus re-modernization, are many and always at work. They include civil society, the critical elites, traditional and social media, influencers, environmental NGOs, subcultures, indigenous minorities, the public sphere, youth (e.g, Greta Thunberg's Fridays for Future), and even “self-organizing psychopaths and counter-experts” (Beck, 2008, p. 81). Movements that initially appeared innocuous such as Greta Thunberg's, who began by skipping school on Fridays to protest against governmental immobility vis-à-vis the climate crisis, can end up mobilizing millions around the world, triggering self-criticism and potentially even green transitions. As the environmental turn occurred during the 1960's−70's proves, under the right conditions, these factors can generate real change in governmental action, potentially leading to RGN and a Green Nation-State in as little as a decade (Conversi and Posocco, 2022). It is true that since the 1970's the world has been through some big transformations, but this does not mean that the present presents less possibilities to trigger a green revolution than there were fifty or 60 years ago.

Institutions especially, such as environmental NGOs and the media that supported the first environmental turn, have been deeply transformed. Moreover, while the surprise effect generated by the first wave of protests didn't give polluters much time to reorganize themselves and react, things are radically different today. There is a fierce resistance facilitated by decades of experience on the parts of those who have strong interests in keeping the status quo i.e., wealthy oil corporations can fund their own research and lobby governments with the aim of denying the climate crisis and fuelling alarmism that if we put a halt to fossil fuels the world would plunge into economic chaos (Chomsky and Pollin, 2020). A recent speech by Australia's PM Anthony Albanese seems to support this claim: “banning fossil fuel exports and new coal and gas mines to try and reduce climate emissions won't stop global warming but would devastate the Australian economy” (The Sydney Morning Herald, 2022).

Australia's PM is arguably not alone in the global political scenario. As Kraft (2001) put it when investigating the case of the US, “Politics increasingly bows to the requirements of economics that demands to “justify environmental policy actions through analysis of economic impacts and through provision of strong scientific analysis such as quantitative risk assessments”” (Kraft, 2001, p. 145). The message has been successfully conveyed worldwide that solving the climate crisis is an extremely difficult task that requires professional figures, biologists, physicists, and economists, to name a few, in organized committees that have the role to suggest good strategies to Nation-States' governments. People are left out of the game. One of the problematic results is that environmental organizations adapted and moved the environmental struggle from the street to the corridors of politics where lobbying is carried out, and the labs of universities and research centers, where environmental studies take shape that lobbies can use to influence governments.

In this scenario, environmental NGOs, whose main role was, not long ago, to act on behalf of and with the people (Edwards, 2020), have politicized and “hyperprofessionalised” (Diani and Donati, 1999). The US is a good example of such a development (Kraft, 2001), but the same happened in Europe (Diani and Donati, 1999). Since the 1980's, slowly but resolutely, many environmental NGOs in the US stopped mobilizing people, organized fewer public protests while using more and more resources to establish roots in Washington DC and lobby governments (Kenner and Heede, 2021). The same is true in the hearth of Europe:

The sudden change in political opportunities available to German environmentalists in the early 1990's […] has indeed exposed the limitations of hyperprofessional groups when their central concerns are not as high on the public agenda as they used to be, their insider status is diminished, and they badly need grass-roots mobilization again. Under deteriorating political conditions, highly professional environmental lobbies might well prove unable to revert to that good, old weapon of excluded interests–contentious protest (Diani and Donati, 1999, p. 30).

It certainly does not mean that environmental organizations did nothing. Even a superficial look at China and Russia, two countries where environmental NGOs are perpetually checked by two of the most powerful authoritarian regimes in the world, shows what the alternative could be. And yet, it would be wrong not to see in the professionalization and politicization of environmental NGOs as a lost opportunity to do better.

**Figure 6:**
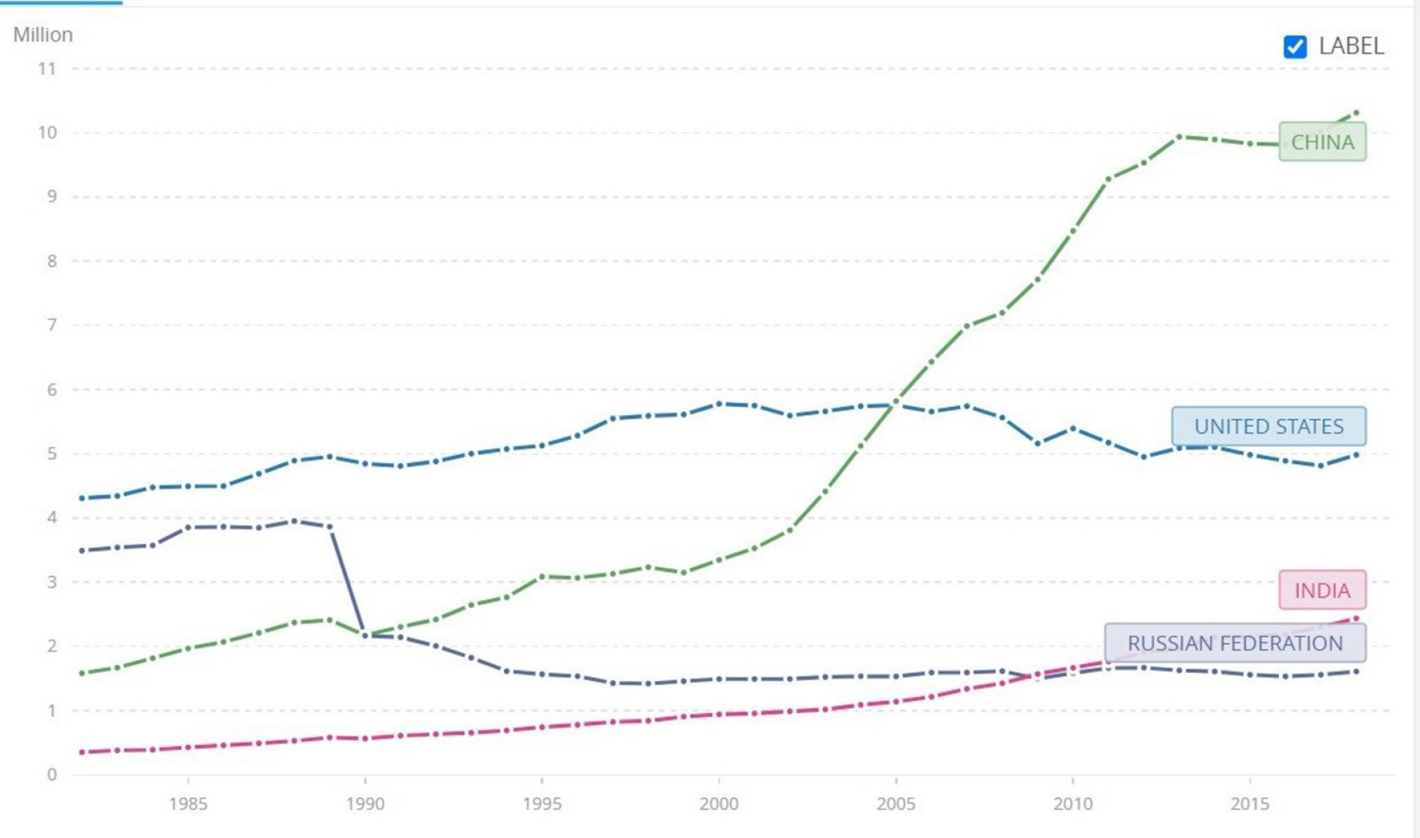
Top-polluting nation-states. CO2 emissions since the 1980's. Source: World Bank. At https://data.worldbank.org/indicator/EN.ATM.CO2E.KT?locations=RU-CN-US (accessed February 26, 2022).

**Figure 7:**
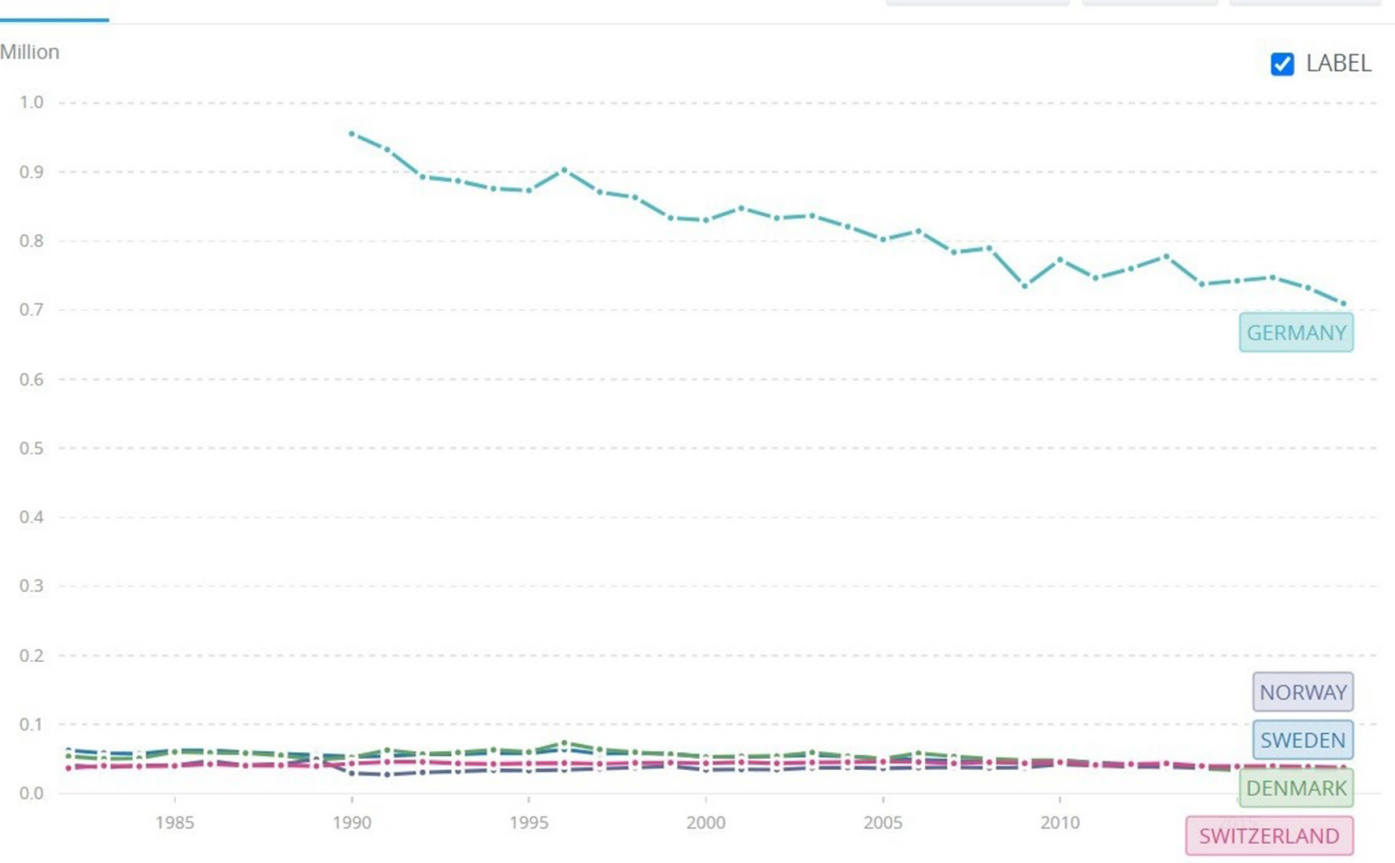
Exemplary nation-states. CO2 emissions since the 1980s. Source: World Bank. At https://data.worldbank.org/indicator/EN.ATM.CO2E.KT?locations=RU-CN-US (accessed February 26, 2022).

Beside badly harming grassroots movements, the politicization of NGOs greatly facilitated the politicization of the climate crisis, which in turn, contributed to an oscillation in terms of environmental policy that strongly undermined the development of reflexive green nationalism. The mechanism is as follows: when a party that seeks to resolve the climate crisis is in power, steps are taken toward that goal and policy is enacted, when climate skeptics or climate deniers rule, they either undo what the previous government did (Donald Trump's government is an example of such a strategy) or do nothing to improve it. This is a process that Cullenward and Victor (2021) recently highlighted, and greatly harms the environment because, unlike elections, the natural world does not work on a 3–4 year basis. To be successful and, say, regenerate devastated and unproductive soil or regrow a forest, environmental policy needs stability in the long run. The same is true for greening the many sectors that emit the most carbon dioxide and other greenhouse gases. This is particularly clear when comparing CO2 emissions in the US and Germany. As Figures 6, 7 (below) show, the curve of emissions in the former goes up and down while the latter is stable and decreases, slowly but resolutely. The “secret” of Germany is that unlike the US, the time of reflexivity didn't stop in the 1970's. It continued well beyond the 1980's until present and spread environmentalism throughout all levels of German society. It gave birth to what we call reflexive green nationalism. If, in the US, the government of Ronald Reagan backtracked, in Germany, the government of Helmut Kohl kept enacting important climate policies, established the Ministry of Environment, Nature Conservation, and Nuclear Safety, and the subcommittee the Enquête Commission on Preventive Measures to Protect the Earth's Atmosphere (Climate Enquête Commission) (Watanabe and Lutz, 2003). The result of the Climate Enquête Commission was instrumental in terms of climate change policies in the future. It set long term goals for emissions reductions, promotion of renewable energy, energy efficiency standards, market-based approaches to climate change, and voluntary agreements with industry that still bear fruit in the present.

A solution to the stagnation of politics and the oscillation of climate policy comes from environmental movements going back to do what they know better: raise awareness of the disastrous effects of climate change, trigger participation and bottom-up mobilization, let people do the work of politics, help them to take to the streets and demonstrate, push politicians to implement climate policy, and control that environmental policy is effectively enforced and functioning. In addition, they should use their networking and symbolic power to echo the work of the scientific community that does not use science for profit but for science's sake. In doing so, they would help correct one of the biggest problems hindering re-modernization, “bad thinking epidemic” (Levy, 2021) that we discussed in the earlier section of this paper, and help people to distinguish between reliable and unreliable sources of information. This is crucial insofar as a populace more sensitive to the cause of environmentalism and more critical makes its voice heard and forces politicians, regardless of their political affiliation, to act when they stand still.

This is a key point. Infuse institutions with values-based energy and direction, and political settlements that legitimize and sustain these values and directions in the polity. Civil society, especially environmental organizations as actors devoted to provide solutions to the climate crisis, should work to depoliticise climate change, not in the sense of pushing climate change out of politics, but to push the idea that, for example, the immediate and drastic cut of CO2 emissions shouldn't be subject of political debate anymore but needs steps that politics must take beyond party flags. Cullenward and Victor (2021) argued that successful climate policy requires building political coalitions to support transforming all the major emitting sectors of the economy, from electric power to transportation. And yet, vis-à-vis the enormous and multifaceted interests in the energy sector, as the case of the US proves, building such coalitions seem to be a gargantuan endeavor. In addition, political coalitions don't guarantee stability in the long run and give birth to oscillation leading to circles of progress and setbacks harming the environment. For the sake of clarity, the goal is not to eradicate political debates or diminish their importance while pushing for green solutions. This would share problematic similarities with eco-fascist developments in the 1920's and 1930's. The goal is to build a deeper environmental consciousness at the large national level, hence the need to tie nationalism and environmentalism. This defined the ideological linkage as Reflexive Green Nationalism, an ideology shared by large sectors of society, including political and economic elites. This would decrease said oscillation and favor green transitions. For this to happen, environmentalism must lock in. The study of green nation-states supports this hypothesis (Conversi and Posocco, 2022; Posocco and Watson, 2022). The lock in of environmentalism, and its entrenchment within the large and deeply rooted nationalist ideology would avoid the climate crisis remaining entangled in petty political debates, coalition games, and political interest.

It is true, the tasks this study envisages for environmental organizations are extremely difficult, and yet their work is arguably much easier today than fifty or so years ago, when they were fundamental in triggering protests that led to the environmental turn. This is also valid in China and Russia, which although remain strongly centralized authoritarian systems, have seen the development (in 1990's Russia one could use the term “flourishing”) of environmental NGOs in ways that were unthinkable fifty years ago. In addition, unlike fifty years ago, both societies are much more open to external influences, including the global market bringing new trends. This plays a decisive role in making them extremely more malleable, as it is clear in China, a country that is already the leading power in green technology, and some argue, also at a turning point in terms of green transition (Heggelund, 2021).

**Figure 8:**
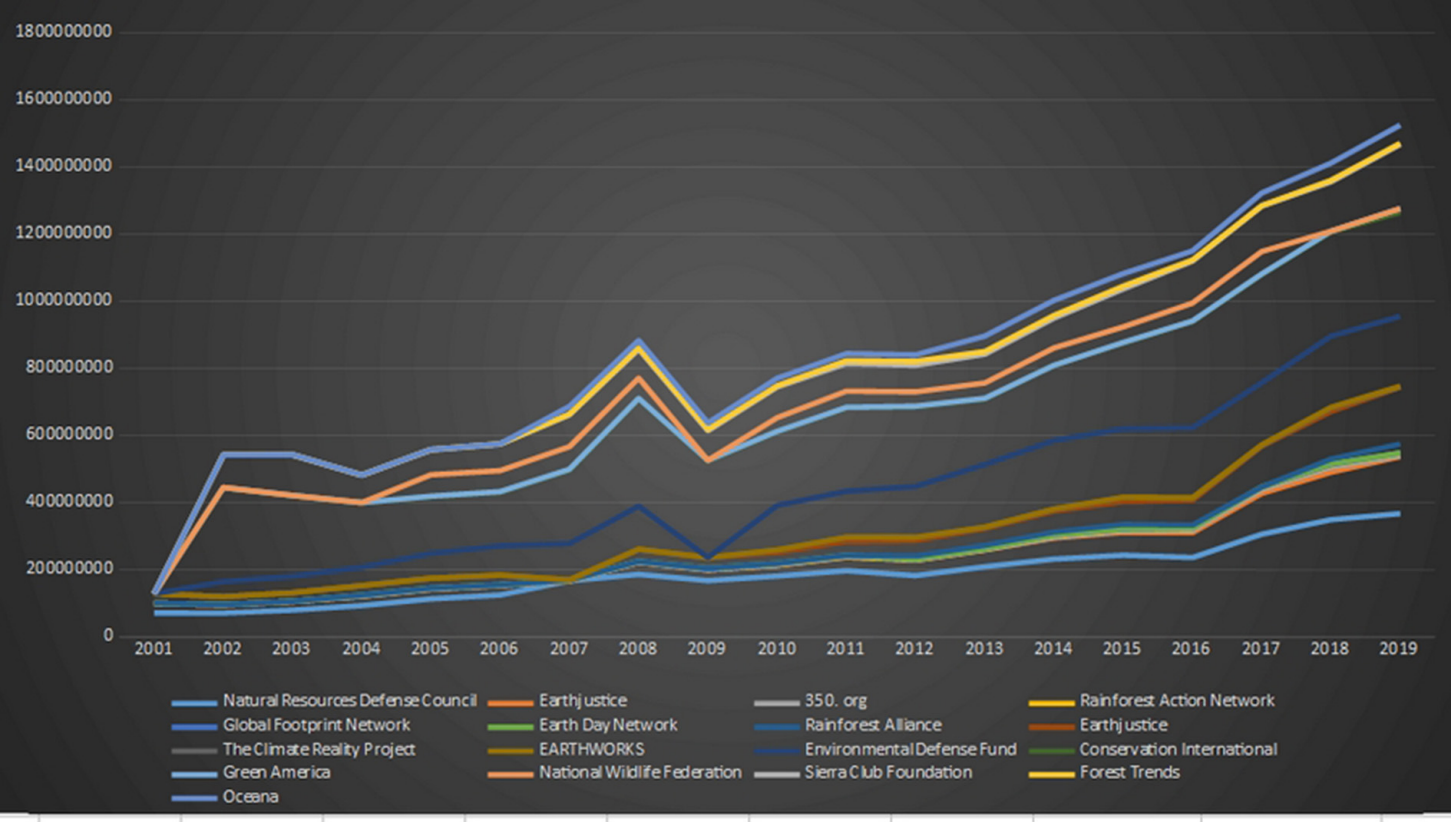
Growth chart of 17 important environmental NGOs (2001–2019).

**Figure 9:**
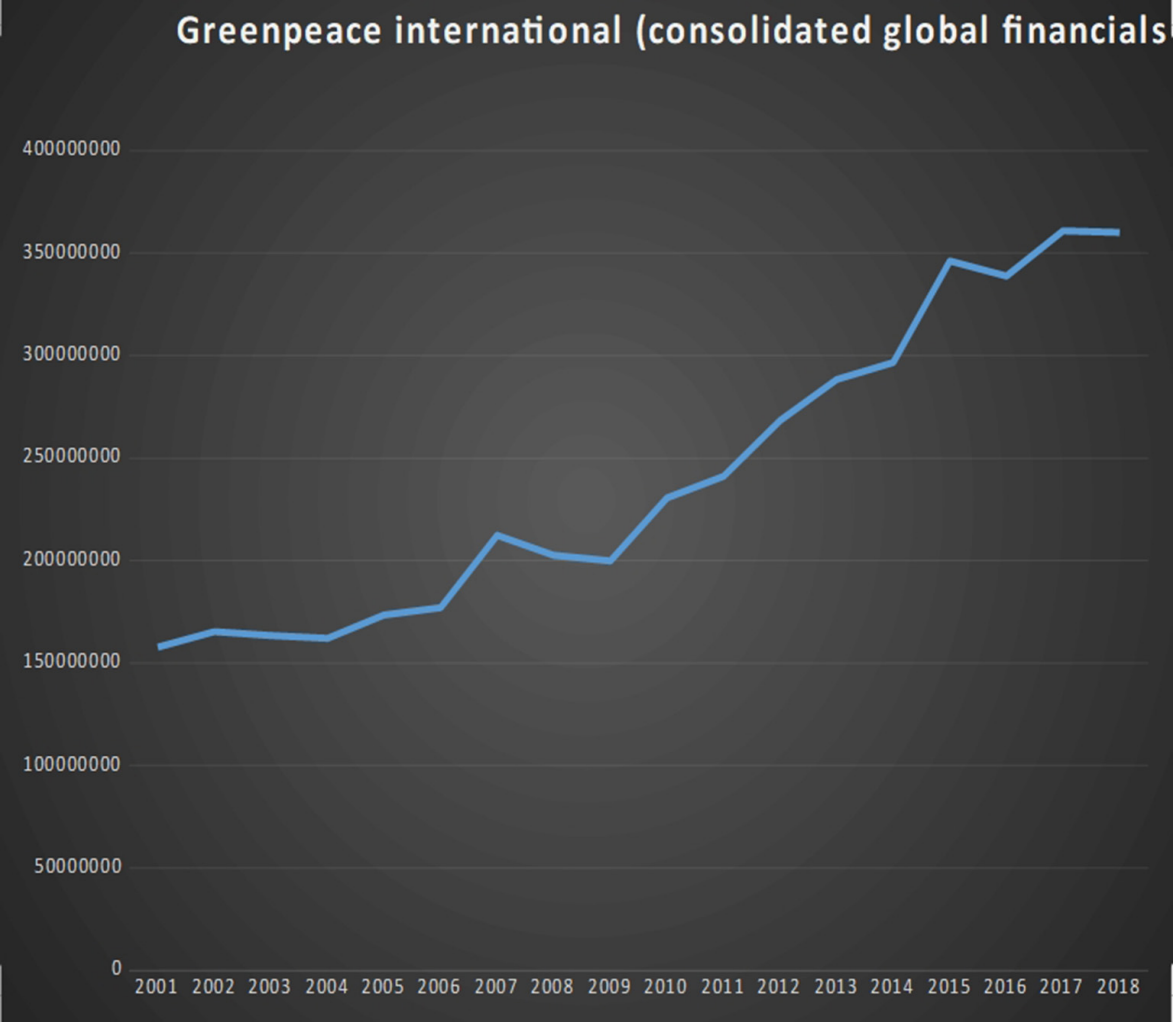
Greenpeace international. Consolidate global financials in dollars. Data source: Greenpeace annual reports. Both are freely accessible from the organisation’s websites.

**Figure 10:**
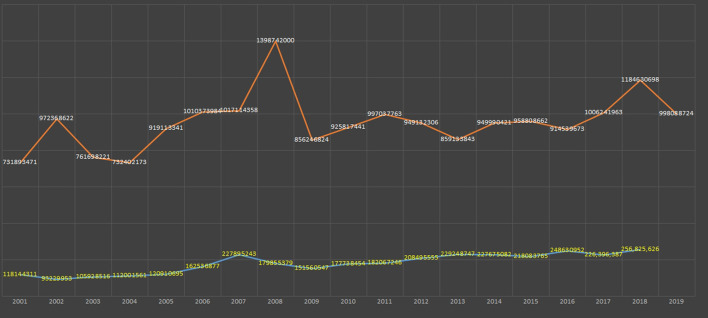
Growth chart of WWF (blue line) and Nature Conservancy (orange line) (2001–2018). Data source: WWF and the Nature Conservancy annual reports. Both are freely accessible from these organisations' websites.

**Figure 11:**
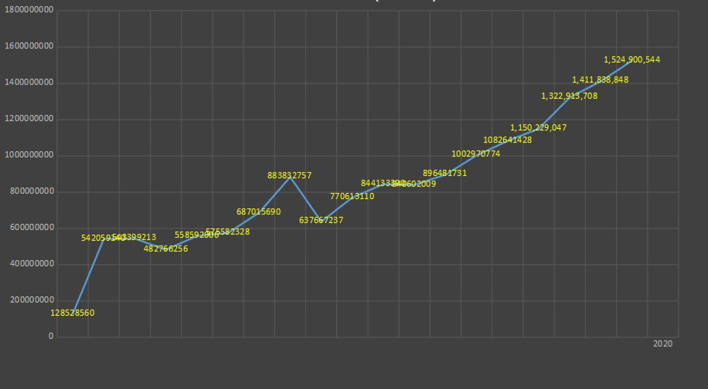
Total net assets of environmental NGOs listed in Figures 8, 9. Year. 2000–2020.

**Figure 12:**
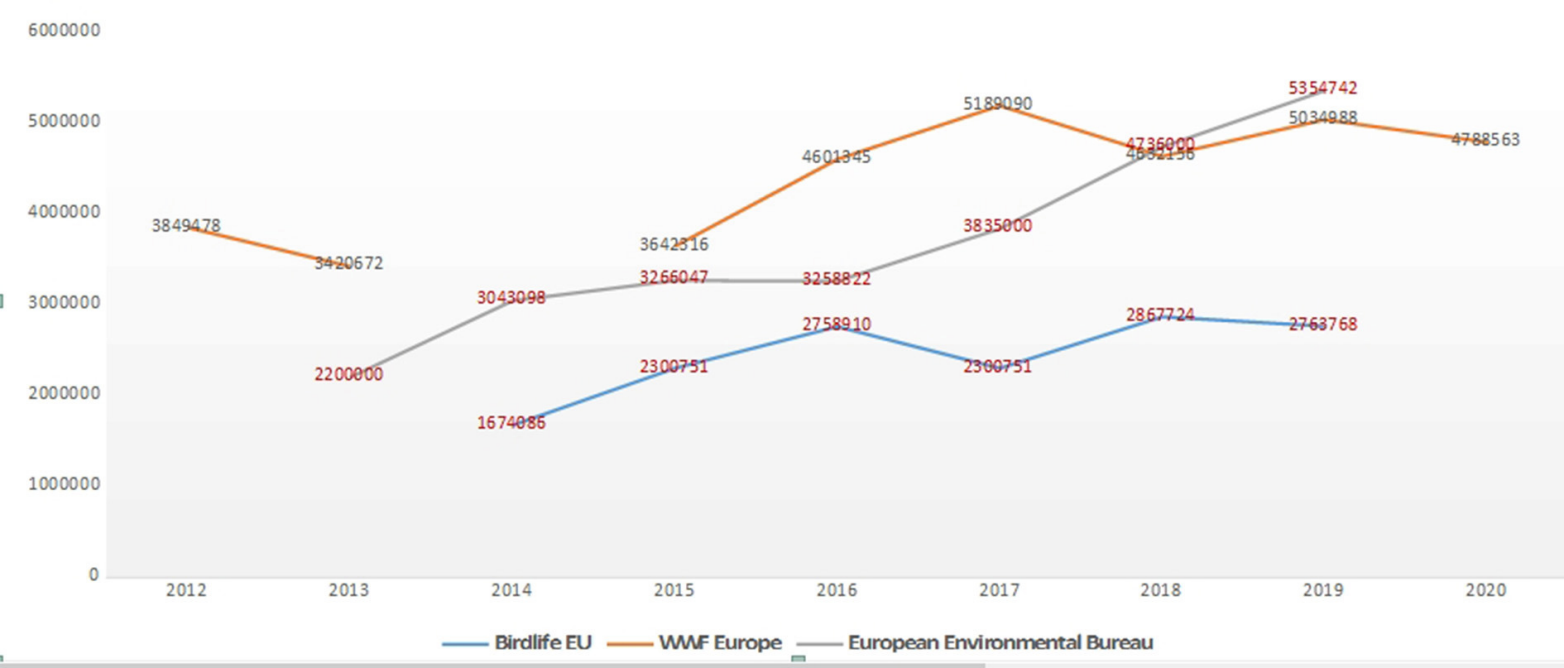
Three Environmental NGOs in Europe. Growth charts. Data source: Birdlife, WWF Europe and European Environmental Bureau annual reports. Freely accessible from these organisations' websites.

Figures 8–12 below show the growth charts of twenty among the biggest environmental organizations around the world, including the World Wildlife Fund (WWF), the Sierra Club Foundation, the Environmental Defense Fund, Rainforest Alliance, and the Nature Conservancy. The graphs show that their total net assets (freely accessible on the websites of these organizations) grew linearly through the last 20 years, and that these institutions are richer, more powerful and potentially more influential than ever.

Environmental NGOs should take advantage of their growing power. As mentioned above, they should implement strategies to depoliticise climate change rather than contributing to its politicization, and should mobilize people and push them to demand the resolution of climate change beyond party flags. This kind of mobilization would provide a strong basis to the development of RGN insofar as citizens that think critically are, as Giddens (1994) put it, “reflexive citizens.” Eventually, as happened in countries such as Germany, which hosts the strongest green party of Europe (BÜNDNIS 90/DIE GRÜNEN), politicians' agendas will adapt to secure those votes. Top-down and bottom-up environmental forces working in concert would give birth, as in Germany, to a virtuous circle that characterizes green nation-states: a synergy between different parts united in making the national society a greener society.

## Conclusion

This article looked at climate change from a novel perspective, the one of nation-state and nationalism. Drawing on theories of nationalism bridged with the theory of reflexive modernity, it explained climate change not as a global problem but as a national one, lying with a few top polluters: nation-states that are incapable of (1) critically reflecting on the problems they create, (2) searching for solutions, and (3) applying them to avoid a global warming that triggers a global catastrophe.

This is a key change in the way we think about the causes of and the available strategies for solving climate change. It suggests an original point of view when considering the larger body of scholarship that emphasize global action. Indeed, this article puts forward the notion that, to resolve the climate crisis, we don't need to change the way humanity thinks about the accelerating pace of energy use, carbon emissions, and fossil fuels. Instead, we need to make sure that top-polluters do. This is true vis-à-vis the overwhelming evidence brought by the 2022 IPCC report that we have no more time. We cannot keep making Pindaric flights theorizing idyllic cosmopolitan or terrestrial futures where all people live harmoniously and face the climate crisis together, coordinated and cooperative. This will not happen within the time frame we are given, and if we don't solve the climate crisis within this time frame, there might be no humanity to build a more “human” world. This is particularly true vis-à-vis Kemp et al.'s study on catastrophic climate change scenarios (Kemp et al., 2022).

Strategies to turn top polluters into green nation-states in the time given by the 2022 IPCC report are need. This article conceptualized “Reflexive Green Nationalism” (RGN) as a potential answer, a national ideology that turns away from uncritical and celebratory forms of nationalism and makes critique of unsustainable national modernization efforts, reflexivity, and search for solutions its founding elements. The conviction of targeting national ideology first came from (1) nationalism studies bringing evidence of the dominance of nationalism and the nation-state in the international political arena, and (2) a recent body of literature on existing green nation-states suggesting that changing the ideas driving a nation to act in and interact with the environment, making citizens more reflexive, critical and ready to mobilize for their national environment, is a fundamental precondition for sustainability. In this perspective, RGN framework and strategies are both pragmatic and normative/ideological. RGN acknowledges both limits and potentials of nationalism, as many others did (Fukuyama, 1992; Gans, 2003; Harari, 2019; Mandelbaum, 2019; Tamir, 2019), and builds on them. Among these elements, also controversial factors such as national emotions, national identity, a sense of belonging to the nation and national pride, factors that are inextricably linked to national homogenization. While the latter is, as Mandelbaum (2019) rightly put it, a “fantasy” of the state giving birth to numerous tensions in multi-ethnic nation-states worldwide, in the context of climate change they become potentially positive and powerful triggers mobilizing the masses. In the end, it is true that the thought of our world “on fire” can be a strong motivation to act, but it is difficult to object that an even stronger motivation comes from knowing that our own house will burn if we don't extinguish the blaze.

This article identified civil society, in particular environmental NGOs, as the key actors with the potential to lead to a change in ideology at the large national level. While we acknowledge previous studies highlighting the shortcomings from such organizations, especially the phenomenon of politicization of environmental organizations (a potential double-edged sword), a comparative look at green nation-states (both in the past and present) shows that these organizations play a key role in creating a healthy epistemic environment, mobilizing masses, letting them do the work of politics, pushing governments to enact climate policy and establishing committees to make sure they are successfully enforced. These are all fundamental factors contributing to the development of green nation-states.

It is also true that environmental organizations are not the only ones playing a role in ideological change. Media and critical elites play similarly important roles. Unfortunately, given the limited space at our disposal, we had to narrow down our focus. We reserve this analysis to a future dedicated article.

This article ends with a discussion on the necessary conditions for developing successful environmental movements considering the problems that these organizations face in the 21st century, especially their politicization within top polluters such as the US, Russia, and China.

## Data availability statement

The raw data supporting the conclusions of this article will be made available by the authors, without undue reservation.

## Author contributions

LP conceived the idea, developed the theory, and wrote the manuscript. IW contributed to the main conceptual ideas, the design of the project, and the writing of the manuscript. All authors contributed to the article and approved the submitted version.

## Conflict of interest

The authors declare that the research was conducted in the absence of any commercial or financial relationships that could be construed as a potential conflict of interest.

## Publisher's note

All claims expressed in this article are solely those of the authors and do not necessarily represent those of their affiliated organizations, or those of the publisher, the editors and the reviewers. Any product that may be evaluated in this article, or claim that may be made by its manufacturer, is not guaranteed or endorsed by the publisher.

## Footnotes

^1^For the sake of clarity, a small number of nation-states within EU28 are among the most dynamic in terms of forms of green transition. These are Sweden, Norway, Denmark, Germany.

^2^Describing nationalist ideologies that see the environment as a resource that humans can exploit, Daniele Conversi (2020) came up with the term Resource Nationalism (RN).

^3^With some expectations, countries such as Turkey and Ukraine seem to have a higher share of emissions, but these countries are the exception rather than the norm.

^4^News that the two were murdered on June 5, 2022 was given by The Guardian. At https://www.theguardian.com/world/ng-interactive/2022/jun/17/the-disappearance-of-dom-phillips-and-bruno-pereira-a-timeline (accessed June 17, 2022).

^5^At https://ccpi.org/.

^6^Norway is a good example of environmentalism directed mostly inwards towards the nation. It is a country that uses almost 100% of renewable energy for domestic needs, but exports massive quantities of oil to other nation-states.
